# Poly[[hexa­aqua­(μ_3_-2,2′-bipyridine-4,4′,6,6′-tetra­carboxyl­ato-κ^6^ *O* ^4^:*N*,*O* ^6^,*O* ^6′^,*N*′:*O* ^4′^)dinickel(II)] dihydrate]

**DOI:** 10.1107/S1600536812043942

**Published:** 2012-10-31

**Authors:** Jie Li, Ke-Wei Lei, Dong-Guo Xia

**Affiliations:** a State Key Lab. Base of Novel Functional Materials and Preparation Science, Institute of Solid Materials Chemistry, Faculty of Materials Science and Chemical Engineering, Ningbo University, Ningbo 315211, People’s Republic of China

## Abstract

In the title complex, {[Ni_2_(C_14_H_4_N_2_O_8_)(H_2_O)_6_]·2H_2_O}_*n*_, the two Ni^II^ atoms are located in different special positions (one on a twofold rotation axis and the second on a centre of symmetry) and have different distorted octa­hedral environments (one by two N atoms from a bipyridine unit, two O atoms from two water mol­ecules and two O atoms from two carboxyl­ate groups, and the second by four O atoms from four water mol­ecules and two O atoms from two carboxyl­ate groups). Thus, the environments of the Ni^II^ atoms may be denoted as NiN_2_O_4_ and NiO_6_. In the crystal, there exists an extensive network of classical O—H⋯O hydrogen bonds.

## Related literature

For the synthesis of title compound, see: Al-Harbi (2011 ▶). 
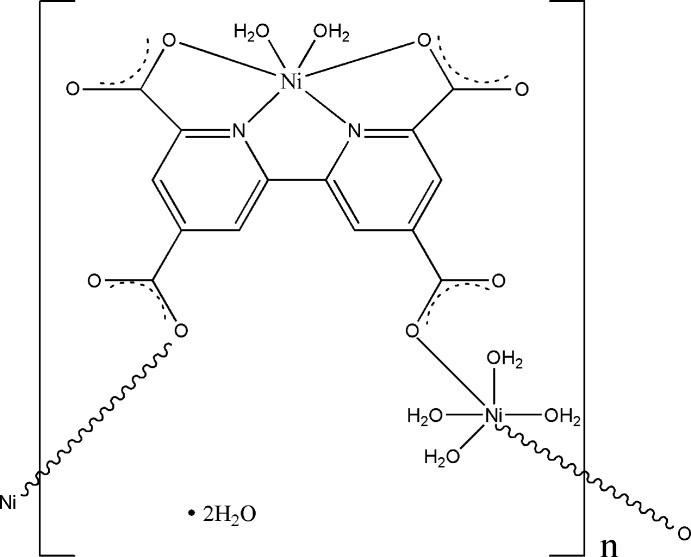


## Experimental

### 

#### Crystal data

[Ni_2_(C_14_H_4_N_2_O_8_)(H_2_O)_6_]·2H_2_O
*M* *_r_* = 589.70
Monoclinic, 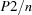

*a* = 7.3588 (8) Å
*b* = 11.8463 (13) Å
*c* = 11.9942 (13) Å
β = 99.184 (1)°
*V* = 1032.19 (19) Å^3^
*Z* = 2
Mo *K*α radiation
μ = 1.91 mm^−1^
*T* = 296 K
0.28 × 0.24 × 0.19 mm

#### Data collection

Rigaku R-AXIS RAPID diffractometer
Absorption correction: multi-scan (*ABSCOR*; Higashi, 1995 ▶) *T*_min_ = 0.591, *T*_max_ = 0.695
8794 measured reflections
2365 independent reflections
2248 reflections with *I* > 2σ(*I*)
*R*_int_ = 0.038

#### Refinement

*R*[*F* ^2^ > 2σ(*F* ^2^)] = 0.023
*wR*(*F* ^2^) = 0.062
*S* = 1.06
2365 reflections
176 parameters
H atoms treated by a mixture of independent and constrained refinement
Δρ_max_ = 0.40 e Å^−3^
Δρ_min_ = −0.42 e Å^−3^

### 

Data collection: *RAPID-AUTO* (Rigaku, 1998 ▶); cell refinement: *RAPID-AUTO*; data reduction: *CrystalStructure* (Rigaku/MSC, 2004 ▶); program(s) used to solve structure: *SHELXS97* (Sheldrick, 2008 ▶); program(s) used to refine structure: *SHELXL97* (Sheldrick, 2008 ▶); molecular graphics: *SHELXTL* (Sheldrick, 2008 ▶); software used to prepare material for publication: *SHELXL97*.

## Supplementary Material

Crystal structure: contains datablock(s) global, I. DOI: 10.1107/S1600536812043942/rk2355sup1.cif

Structure factors: contains datablock(s) I. DOI: 10.1107/S1600536812043942/rk2355Isup2.hkl

Additional supplementary materials:  crystallographic information; 3D view; checkCIF report

## Figures and Tables

**Table 1:** Hydrogen-bond geometry (Å, °)

*D*—H⋯*A*	*D*—H	H⋯*A*	*D*⋯*A*	*D*—H⋯*A*
O3—H3*B*⋯O6	0.85 (2)	1.95 (2)	2.7821 (16)	169 (2)
O3—H3*A*⋯O8^i^	0.82	1.90	2.7189 (16)	174
O4—H4*A*⋯O6^ii^	0.82	1.95	2.7697 (17)	178
O4—H4*B*⋯O7^iii^	0.78 (3)	2.03 (3)	2.7997 (16)	172 (3)
O5—H5*A*⋯O2^iv^	0.82	1.90	2.6891 (16)	162
O5—H5*B*⋯O7^v^	0.78 (3)	1.96 (3)	2.7395 (17)	172 (3)
O6—H6*A*⋯O2^v^	0.79 (3)	2.03 (3)	2.8096 (17)	170 (2)

Symmetry codes: (i) 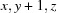; (ii) 
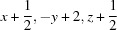
; (iii) 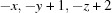; (iv) 
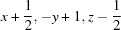
; (v) 
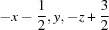
.

**Fig. 1.**
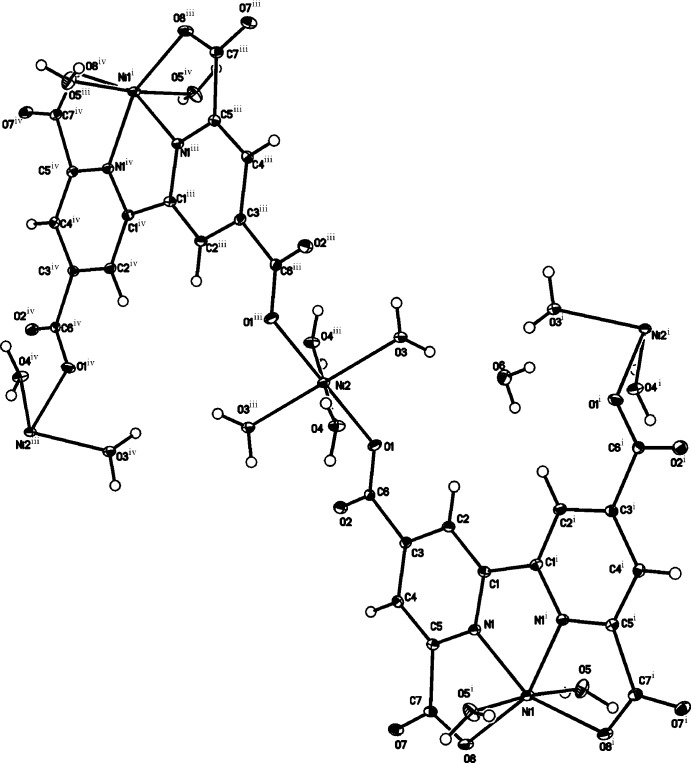
The structure of the title complex with the atom–numbering scheme. Displacement ellipsoids are drawn at 30% probability level.

**Fig. 2.**
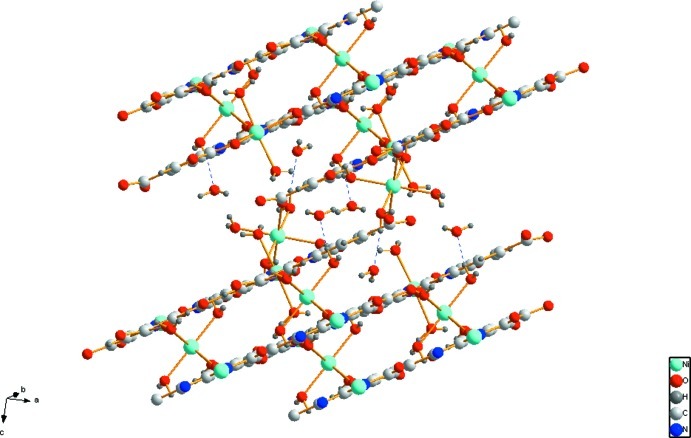
The three–dimensional structure of the title complex. The hydrogen bonds are indicated by dashed lines.

## supplementary crystallographic information

### Comment

The complex structure is shown in Fig. 1. In the title complex two Ni atoms are placed in two different special positions: Ni1 - on 2–fold axis and Ni2 - in centre of symmetry. These atoms have different environment: Ni1 is coordinated by two N atoms of dipyridine moiety, two O atoms from water molecules and two O atoms from two carboxylate moieties; Ni2 is coordinated only by six O atoms: four O from four water molecules and two O from two carboxylate moieties.

In the crystal structure there is the wide net of classical O—H···O type H–bonds (Table 1, Fig. 2), which stabilize crystal packing.

### Experimental

A mixture of 6–(4,6–dicarboxypyridin–2–yl)pyridine–2,4–dicarboxylic acid (0.0332 g, 0.1 mmol), Ni(NO_3_)_2_^.^6H_2_O (0.0724 g, 0.3 mmol) and water (10 ml) was placed in a teflon–lined stainless steel vessel (25 ml) and heated at 443.15 K for 72 h, and then cooled to room temperature at a rate of 5/h (Al-Harbi, 2011). The resulting green single crystals were isolated by washing with *DMF* and dried *in vacuo*. Yield: 62.3%.

### Refinement

All H atoms were placed in geometrically idealized positions and constrained to ride on their parent atoms (C—H = 0.93%A; N—H = 0.86Å; O—H = 0.82Å) and *U*_iso_(H) values weren taken to be equal to 1.2 *U*_eq_(C, N) and 1.5*U*_eq_(O).

### Crystal data

**Table:**

[Ni_2_(C_14_H_4_N_2_O_8_)(H_2_O)_6_]·2H_2_O	*F*(000) = 604
*M**_r_* = 589.70	*D*_x_ = 1.897 Mg m^−^^3^
Monoclinic, *P*2/*n*	Mo *K*α radiation, λ = 0.71073 Å
Hall symbol: -P 2yac	Cell parameters from 8794 reflections
*a* = 7.3588 (8) Å	θ = 1.7–27.5°
*b* = 11.8463 (13) Å	µ = 1.91 mm^−^^1^
*c* = 11.9942 (13) Å	*T* = 296 K
β = 99.184 (1)°	Block, green
*V* = 1032.19 (19) Å^3^	0.28 × 0.24 × 0.19 mm
*Z* = 2	

### Data collection

**Table:**

Rigaku R-AXIS RAPID diffractometer	2365 independent reflections
Radiation source: fine–focus sealed tube	2248 reflections with *I* > 2σ(*I*)
Graphite monochromator	*R*_int_ = 0.038
Detector resolution: 0 pixels mm^-1^	θ_max_ = 27.5°, θ_min_ = 1.7°
ω scans	*h* = −9→8
Absorption correction: multi-scan (*ABSCOR*; Higashi, 1995)	*k* = −15→14
*T*_min_ = 0.591, *T*_max_ = 0.695	*l* = −15→15
8794 measured reflections	

### Refinement

**Table:**

Refinement on *F*^2^	Primary atom site location: structure-invariant direct methods
Least-squares matrix: full	Secondary atom site location: difference Fourier map
*R*[*F*^2^ > 2σ(*F*^2^)] = 0.023	Hydrogen site location: inferred from neighbouring sites
*wR*(*F*^2^) = 0.062	H atoms treated by a mixture of independent and constrained refinement
*S* = 1.06	*w* = 1/[σ^2^(*F*_o_^2^) + (0.0255*P*)^2^ + 0.5729*P*] where *P* = (*F*_o_^2^ + 2*F*_c_^2^)/3
2365 reflections	(Δ/σ)_max_ < 0.001
176 parameters	Δρ_max_ = 0.40 e Å^−^^3^
0 restraints	Δρ_min_ = −0.42 e Å^−^^3^

### Special details

**Table:**

Geometry. All s.u.'s (except the s.u. in the dihedral angle between two l.s. planes) are estimated using the full covariance matrix. The cell s.u.'s are taken into account individually in the estimation of s.u.'s in distances, angles and torsion angles; correlations between s.u.'s in cell parameters are only used when they are defined by crystal symmetry. An approximate (isotropic) treatment of cell s.u.'s is used for estimating s.u.'s involving l.s. planes.
Refinement. Refinement of *F*^2^ against ALL reflections. The weighted *R*–factor *wR* and goodness of fit *S* are based on *F*^2^, conventional *R*–factors *R* are based on *F*, with *F* set to zero for negative *F*^2^. The threshold expression of *F*^2^ > σ(*F*^2^) is used only for calculating *R*–factors(gt) *etc*. and is not relevant to the choice of reflections for refinement. *R*–factors based on *F*^2^ are statistically about twice as large as those based on *F*, and *R*–factors based on ALL data will be even larger.

### Fractional atomic coordinates and isotropic or equivalent isotropic displacement parameters (Å^2^)

**Table:**

	*x*	*y*	*z*	*U*_iso_*/*U*_eq_	
Ni1	0.2500	0.36726 (2)	0.7500	0.01154 (8)	
Ni2	0.0000	1.0000	1.0000	0.01064 (8)	
O1	−0.10038 (16)	0.87338 (9)	0.89420 (9)	0.0161 (2)	
O3	0.10695 (15)	1.06255 (9)	0.86383 (9)	0.0145 (2)	
H3A	0.0846	1.1302	0.8571	0.022*	
O2	−0.33996 (15)	0.76758 (9)	0.92552 (9)	0.0162 (2)	
O4	0.23852 (15)	0.90408 (10)	1.03816 (9)	0.0162 (2)	
H4A	0.3218	0.9436	1.0709	0.024*	
O8	0.04444 (15)	0.28697 (9)	0.82901 (9)	0.0152 (2)	
C1	0.1670 (2)	0.60269 (12)	0.77858 (12)	0.0118 (3)	
N1	0.10800 (18)	0.49784 (10)	0.79747 (10)	0.0110 (2)	
C6	−0.1837 (2)	0.78123 (12)	0.89848 (11)	0.0112 (3)	
C4	−0.1418 (2)	0.56825 (12)	0.88022 (11)	0.0112 (3)	
H4C	−0.2463	0.5552	0.9130	0.013*	
C2	0.0741 (2)	0.69601 (13)	0.81220 (12)	0.0123 (3)	
H2A	0.1153	0.7688	0.8011	0.015*	
C5	−0.0391 (2)	0.47890 (12)	0.84663 (12)	0.0112 (3)	
C3	−0.0820 (2)	0.67854 (12)	0.86283 (11)	0.0108 (3)	
O7	−0.21194 (15)	0.32298 (9)	0.90173 (9)	0.0157 (2)	
O5	0.07941 (17)	0.35897 (11)	0.59723 (10)	0.0201 (2)	
H5A	0.1245	0.3169	0.5548	0.030*	
C7	−0.0751 (2)	0.35285 (12)	0.86043 (12)	0.0120 (3)	
O6	0.02584 (17)	0.96743 (11)	0.64958 (10)	0.0191 (2)	
H6A	−0.015 (3)	0.909 (2)	0.6259 (19)	0.031 (6)*	
H6B	0.114 (4)	0.976 (2)	0.621 (2)	0.037 (7)*	
H5B	−0.025 (4)	0.352 (2)	0.603 (2)	0.043 (7)*	
H4B	0.240 (4)	0.842 (2)	1.059 (2)	0.047 (8)*	
H3B	0.069 (3)	1.031 (2)	0.801 (2)	0.035 (6)*	

### Atomic displacement parameters (Å^2^)

**Table:**

	*U*^11^	*U*^22^	*U*^33^	*U*^12^	*U*^13^	*U*^23^
Ni1	0.01010 (14)	0.00796 (14)	0.01721 (14)	0.000	0.00415 (10)	0.000
Ni2	0.01066 (15)	0.00722 (14)	0.01465 (14)	0.00026 (9)	0.00393 (10)	−0.00035 (9)
O1	0.0192 (6)	0.0099 (5)	0.0203 (5)	−0.0022 (4)	0.0067 (4)	−0.0040 (4)
O3	0.0182 (6)	0.0093 (5)	0.0165 (5)	−0.0002 (4)	0.0048 (4)	0.0003 (4)
O2	0.0151 (5)	0.0139 (5)	0.0209 (5)	0.0015 (4)	0.0067 (4)	0.0015 (4)
O4	0.0140 (5)	0.0103 (5)	0.0241 (5)	0.0009 (4)	0.0025 (4)	0.0019 (4)
O8	0.0136 (5)	0.0090 (5)	0.0239 (5)	0.0007 (4)	0.0059 (4)	0.0014 (4)
C1	0.0120 (7)	0.0097 (7)	0.0135 (6)	−0.0012 (5)	0.0020 (5)	0.0007 (5)
N1	0.0108 (6)	0.0092 (6)	0.0131 (6)	−0.0003 (4)	0.0025 (5)	−0.0002 (4)
C6	0.0130 (7)	0.0107 (7)	0.0097 (6)	0.0016 (5)	0.0015 (5)	0.0008 (5)
C4	0.0098 (7)	0.0124 (7)	0.0114 (6)	−0.0002 (5)	0.0018 (5)	0.0010 (5)
C2	0.0138 (7)	0.0092 (7)	0.0140 (6)	−0.0012 (5)	0.0023 (5)	0.0005 (5)
C5	0.0106 (7)	0.0107 (7)	0.0119 (6)	−0.0004 (5)	0.0005 (5)	0.0008 (5)
C3	0.0112 (7)	0.0100 (7)	0.0109 (6)	0.0003 (5)	0.0001 (5)	−0.0010 (5)
O7	0.0127 (5)	0.0124 (5)	0.0227 (5)	−0.0007 (4)	0.0049 (4)	0.0029 (4)
O5	0.0117 (6)	0.0275 (7)	0.0217 (6)	−0.0010 (5)	0.0045 (4)	−0.0089 (5)
C7	0.0111 (7)	0.0103 (7)	0.0139 (6)	0.0001 (5)	−0.0004 (5)	0.0012 (5)
O6	0.0156 (6)	0.0219 (6)	0.0210 (6)	−0.0053 (5)	0.0061 (5)	−0.0033 (5)

### Geometric parameters (Å, º)

**Table:**

Ni1—N1^i^	1.9996 (12)	O8—C7	1.2769 (18)
Ni1—N1	1.9996 (12)	C1—N1	1.3468 (18)
Ni1—O5	2.0516 (12)	C1—C2	1.393 (2)
Ni1—O5^i^	2.0516 (12)	C1—C1^i^	1.493 (3)
Ni1—O8	2.1327 (11)	N1—C5	1.3317 (19)
Ni1—O8^i^	2.1327 (11)	C6—C3	1.525 (2)
Ni2—O1	2.0265 (11)	C4—C5	1.397 (2)
Ni2—O1^ii^	2.0265 (11)	C4—C3	1.405 (2)
Ni2—O3^ii^	2.0607 (10)	C4—H4C	0.9300
Ni2—O3	2.0607 (10)	C2—C3	1.398 (2)
Ni2—O4^ii^	2.0796 (11)	C2—H2A	0.9300
Ni2—O4	2.0796 (11)	C5—C7	1.530 (2)
O1—C6	1.2570 (18)	O7—C7	1.2427 (19)
O3—H3A	0.8200	O5—H5A	0.8200
O3—H3B	0.85 (2)	O5—H5B	0.78 (3)
O2—C6	1.2541 (18)	O6—H6A	0.79 (3)
O4—H4A	0.8200	O6—H6B	0.79 (3)
O4—H4B	0.78 (3)		
			
N1^i^—Ni1—N1	78.65 (7)	H3A—O3—H3B	108.0
N1^i^—Ni1—O5	93.20 (5)	Ni2—O4—H4A	109.5
N1—Ni1—O5	91.05 (5)	Ni2—O4—H4B	124 (2)
N1^i^—Ni1—O5^i^	91.05 (5)	H4A—O4—H4B	114.4
N1—Ni1—O5^i^	93.20 (5)	C7—O8—Ni1	115.42 (9)
O5—Ni1—O5^i^	174.51 (7)	N1—C1—C2	119.82 (13)
N1^i^—Ni1—O8	155.70 (5)	N1—C1—C1^i^	112.73 (8)
N1—Ni1—O8	77.21 (5)	C2—C1—C1^i^	127.45 (8)
O5—Ni1—O8	89.96 (5)	C5—N1—C1	122.43 (12)
O5^i^—Ni1—O8	87.60 (5)	C5—N1—Ni1	119.63 (10)
N1^i^—Ni1—O8^i^	77.21 (5)	C1—N1—Ni1	117.94 (10)
N1—Ni1—O8^i^	155.70 (5)	O2—C6—O1	126.58 (14)
O5—Ni1—O8^i^	87.60 (5)	O2—C6—C3	118.75 (13)
O5^i^—Ni1—O8^i^	89.96 (5)	O1—C6—C3	114.64 (13)
O8—Ni1—O8^i^	127.03 (6)	C5—C4—C3	117.70 (13)
O1—Ni2—O1^ii^	180.00 (4)	C5—C4—H4C	121.1
O1—Ni2—O3^ii^	94.76 (4)	C3—C4—H4C	121.1
O1^ii^—Ni2—O3^ii^	85.24 (4)	C1—C2—C3	118.91 (13)
O1—Ni2—O3	85.24 (4)	C1—C2—H2A	120.5
O1^ii^—Ni2—O3	94.76 (4)	C3—C2—H2A	120.5
O3^ii^—Ni2—O3	180.000 (1)	N1—C5—C4	121.05 (13)
O1—Ni2—O4^ii^	93.22 (5)	N1—C5—C7	112.28 (12)
O1^ii^—Ni2—O4^ii^	86.78 (5)	C4—C5—C7	126.67 (13)
O3^ii^—Ni2—O4^ii^	87.43 (4)	C2—C3—C4	120.06 (13)
O3—Ni2—O4^ii^	92.57 (4)	C2—C3—C6	118.55 (13)
O1—Ni2—O4	86.78 (5)	C4—C3—C6	121.39 (13)
O1^ii^—Ni2—O4	93.22 (5)	Ni1—O5—H5A	109.5
O3^ii^—Ni2—O4	92.57 (4)	Ni1—O5—H5B	113.3 (19)
O3—Ni2—O4	87.43 (4)	H5A—O5—H5B	119.4
O4^ii^—Ni2—O4	180.0	O7—C7—O8	125.72 (14)
C6—O1—Ni2	138.47 (10)	O7—C7—C5	119.10 (13)
Ni2—O3—H3A	109.5	O8—C7—C5	115.18 (13)
Ni2—O3—H3B	115.5 (17)	H6A—O6—H6B	105 (2)

Symmetry codes: (i) −*x*+1/2, *y*, −*z*+3/2; (ii) −*x*, −*y*+2, −*z*+2.

### Hydrogen-bond geometry (Å, º)

**Table:**

*D*—H···*A*	*D*—H	H···*A*	*D*···*A*	*D*—H···*A*
O3—H3*B*···O6	0.85 (2)	1.95 (2)	2.7821 (16)	169 (2)
O3—H3*A*···O8^iii^	0.82	1.90	2.7189 (16)	174
O4—H4*A*···O6^iv^	0.82	1.95	2.7697 (17)	178
O4—H4*B*···O7^v^	0.78 (3)	2.03 (3)	2.7997 (16)	172 (3)
O5—H5*A*···O2^vi^	0.82	1.90	2.6891 (16)	162
O5—H5*B*···O7^vii^	0.78 (3)	1.96 (3)	2.7395 (17)	172 (3)
O6—H6*A*···O2^vii^	0.79 (3)	2.03 (3)	2.8096 (17)	170 (2)

Symmetry codes: (iii) *x*, *y*+1, *z*; (iv) *x*+1/2, −*y*+2, *z*+1/2; (v) −*x*, −*y*+1, −*z*+2; (vi) *x*+1/2, −*y*+1, *z*−1/2; (vii) −*x*−1/2, *y*, −*z*+3/2.
